# A revitalized biopsychosocial model: core theory, research paradigms, and clinical implications

**DOI:** 10.1017/S0033291723002660

**Published:** 2023-12

**Authors:** Derek Bolton

**Affiliations:** Department of Psychology, Institute of Psychiatry, Psychology and Neuroscience, King's College London, London, UK

**Keywords:** biomedical model, biopsychosocial model, mind–body, psychiatry, regulatory mechanisms

## Abstract

The biopsychosocial model (BPSM) was proposed by George Engel in 1977 as an improvement to the biomedical model (BMM), to take account of psychological and social as well as biological factors relevant to health and disease. Since then the BPSM has had a mixed reputation, as the overarching framework for psychiatry, perhaps for medicine generally, while also being criticized for being theoretically and empirically vacuous. Over the past few decades, substantial evidence has accumulated supporting the BPSM but its theory remains less clear. The first part of this paper reviews recent well-known, general theories in the relevant sciences that can provide a theoretical framework of the model, constituting a revitalized BPSM capable of theorizing causal interactions within and between biological, psychological, and social domains. Fundamental concepts in this new framework include causation as regulation and dysfunction as dysregulation. Associated research paradigms are outlined in Part 2. Research in psychological therapies and social epidemiology are major examples of programs that have produced results anomalous for the BMM and consistent with the BPSM. Theorized models of causal mechanisms enrich empirical data and two biopsychosocial examples are models of chronic stress and pain perception. Clinical implications are reviewed in Part 3. The BPSM accommodates psychological and social as well as biological treatment effects evident in the clinical trials literature. Personal, interpersonal, and institutional aspects of clinical care are out of the scope of the BMM, assigned to the art of healthcare rather than the science, but can be accommodated and theorized in the BPSM.

## Introduction: the problem area and a proposal

The biopsychosocial model (BPSM) was proposed by George Engel in 1977 as an improvement to the biomedical model (BMM), necessary to account for psychological and social factors in health and disease as well as biological (Engel, 1977). This proposal remains critical in science and in service planning (Wade & Halligan, 2017). However, the BPSM has a mixed reputation: it has been regarded as the overarching, dominant framework for psychiatry, perhaps for medicine generally, while on the other hand, and often at the same time, it has been roundly criticized for being scientifically, theoretically vacuous (Ghaemi, 2009; McManus, 2005). This is the problem area to be addressed in this paper: what is the science of the BPSM?

At the level of the empirical data, there is reason to think now that this supports the broad BPSM. Over the past few decades, substantial evidence has accumulated that psychosocial as well as biological factors are implicated in the etiology and course of a wide range of health conditions, supporting the BPSM, reviewed, for example, in Novack et al. (2007), Bolton and Gillett (2019), and in a recent edited volume on biopsychosocial psychiatry (Savulescu, Roache, Davies, & Loebel, 2020). There remains, however, the problem of theory, identified by authoritative critics as cited above: the BPSM seems to have no clear scientific theoretical content. Put another way, the criticisms of the BPSM are typically not reviews of the evidence base, but are doubts about whether the ‘model’ really has anything to say – any content.

Drawing on previous work (Bolton & Gillett, 2019), I will present a case that Engel's main idea – that a BPSM was required to replace the BMM – was visionary but programmatic. It was visionary in anticipating radical changes in the ways that health and disease were becoming theorized and researched, but programmatic because the radical changes were in their early stages, still in progress and not yet widely implemented. However, I suggest, the position has changed by now, and theories that can underpin a broader BPSM are well-known and can be drawn upon to revitalize the model.

To explain the background and the proposal further, Engel's 1977 paper implicates two foundational underpinnings of the BMM, the model for biomedicine as conceived at that time: one is that biology, as physiology, is reducible to physics and chemistry, and the other is the assumption of mind/body dualism (Engel, 1977, p. 379). Dualism notoriously offers no scientific account of how immaterial mental processes can causally influence the material body, and, with this assumption, there is no role for psychological causes in the scientific explanation of behavior unless they are somehow reducible to biological causes. Engel implies that these two foundational underpinnings of the BMM will be abandoned. As to what the replacements are and how they work in a new BPSM, Engel refers to von Bertallanfy's then relatively new General System Theory (Engel, 1977, pp. 391–392; Von Bertalanffy, 1968), but there is no detailed account. However, there were paradigm shifts underway in the biological and psychological sciences at around that time, the emergence of information-based models in biology and the so-called cognitive revolution in psychology, which subsequently have become mainstream science. I will present a case that these new paradigms changed the reductionist assumptions that Engel attributed to the BMM, paving the way for theoretical content for the BPSM.

A defining feature of the BPSM is its interdisciplinarity. High levels of interdisciplinarity require a unified theoretical perspective and integration around shared themes and questions (Boden, 1999; Committee on Facilitating Interdisciplinary Research, 2004; Strijbos, 2010). For the BPSM, shared themes and questions are straightforwardly specifiable about the causes and cures of illness. The substantial task for the BPSM is to explicate a unified theoretical perspective and integration across the three relevant sciences. It turns out, I will propose in what follows, that the required shared theoretical perspectives is systems theoretic, as Engel anticipated, in which concepts such as regulation and control, information and communication, function and dysfunction, play critical roles across the whole biopsychosocial domain.

To make the case for the proposal that the BPSM can be revitalized in terms of current scientific theory I will review, in Part 1, well-known general theories in the three relevant sciences, associated with many research groups, which can provide theoretical content to the model. None of these well-known general theories are, I believe, currently controversial; their competitors are not so much in the current science but in the paradigms being replaced, particularly versions of biological reductionism. In this sense, the revitalized version of the BPSM outlined here is, I suggest, a plausible representation of an overarching model currently in the health sciences. Research paradigms and examples of research programs that implicate the broad range of biopsychosocial factors and which are consistent with the BPSM (regardless whether or not they explicitly invoke the model), are considered in Part 2, and clinical implications will be considered in Part 3.

## BPSM core theory

### The new biology

Engel supposed that the BMM supposed biology was or was reducible to physics and chemistry (Engel, 1977). This reductionist supposition was pretty much orthodoxy at that time, but is now history. Biology (as physiology) has transformed itself in the last few decades, in a revolution largely driven in fact by biomedicine. Biology (as physiology), with biomedicine, is now a closely integrated combination of two kinds of science: one is the physics and chemistry of energy exchanges and transformations (quantified in enthalpy change equations, for example), but there is in addition something new: models of mechanisms for the regulation and control of the physics and chemistry, and of other systems, maintaining functions, typically using feedback by information-transfer. The new ‘regulatory’ paradigm, also known as the ‘information-processing’ revolution, appeared clearly in molecular biology in the 1960s and 1970s, such as Jacob and Monod's ground-breaking work on genetic regulatory mechanisms in the synthesis of proteins (Jacob & Monod, 1961; Lewis, 2013).

The new paradigm in biology can be dated to the physicist Ernst Schrödinger (1944) ground-breaking definition of life as local areas in which the overall direction of the 2nd law of thermodynamics is reversed – life decreases entropy, temporarily (Morange, 2020; Schrödinger, 1944). Schrödinger saw that this would have to involve control of energy production, and he further hypothesized that this was done by genes. An intellectual pathway can be traced from Schrödinger's new conceptualization of biological systems to von Bertalanffy's general system theory (Von Bertalanffy, 1968) to Engel's 1997 paper. Von Bertalanffy proposed that negative entropy was achieved in open systems, in biology and in wider domains including the psychological and the social, and Engel refers to von Bertalanffy's general system theory as a key theoretical driver for the new BPSM, anticipating impacts on healthcare science and practice (Engel, 1977, pp. 391–392). The paradigm shift was, however, still in the early stages in the 1970s – the detailed work in theory development and new associated research paradigms has been underway in the decades since and continues.

The regulatory mechanisms that are central in the new biology have several core features that change the theoretical foundations of the life sciences in ways critical to explicating the BPSM. First, they are causal, but they are not, and are not reducible to, the energy-related equations of physics and chemistry. Second, and connected, regulatory mechanisms can break down, allowing foundational distinctions between life and death, health and disease, that are unavailable in physics and chemistry. Third, the same kind of theoretical apparatus used in biology (function, organization, regulation and dysregulation, information, production, and distribution) is also used in the psychological and social sciences – as reviewed below.

### The new psychology

As noted in the Introduction, Engel supposed that the BMM assumed body/mind dualism and that this was an obstacle to accounting for psychological factors in health and disease. I outline in this section two general ways in which post-dualist, cross-disciplinary theories have been developed over recent decades, critical to formulating a biopsychological model.

Post-dualist models can be characterized as having two working assumptions: one is that mental processes regulate behavior and the other is that mental processing is a function of brain processing. The first point underpins cognitive psychology, while the second merges psychology with neuroscience. Both points are connected with the theoretical innovations in biology in the 1960s and 1970s outlined in the previous section: biology, psychology, and neuroscience all shared interests in new information-based models of the regulation of biological systems and behavior – the paradigm shift went across the life sciences.

The clearest expression of dualist assumptions in psychology was in behaviorism, which explicitly excluded mental processes from explanations of behavior – a position much like Engel attributed to the BMM. From around the 1960s onwards, however, behaviorism was swept away in the cognitive revolution (Miller, 2003; Xiong & Proctor, 2018).

The cognitive revolution influenced many specialties in psychology, not only learning theory. Cognitive models in clinical psychology emphasized the causal role of personal beliefs in the regulation of affect and behavior, such as Beck's cognitive model of depression (Beck, Rush, Shaw, & Emery, 1979). Further, there emerged around this period a class of psychological models focused on the role of expectancies and beliefs about personal control over events – or personal agency – and their implications for well-being. Main examples include Julian Rotter's locus of control theory (Rotter, 1966), Martin Seligman and colleagues’ learned helplessness theory (Seligman & Maier, 1967) and associated model of depression (Miller & Seligman, 1975), Albert Bandura's self-efficacy theory (Bandura, 1982, 2006), and Richard Lazarus and Susan Folkman's work on stress, appraisal, and coping (Lazarus & Folkman, 1984).

Importantly, this class of models spanned many psychology specialty areas, across many domains – physiology, learning, personality, and social – and interactions between them. In this sense, they already constituted a theorized BPSM within the broad psychological tent.

The appearance of personal processes in the new psychological science – beliefs, about the world and our agency, personal goals, emotions, and behavior – has substantial relevance to the question whether a broader BPSM is needed in health science and healthcare. Engel gave a long list of important issues the BMM could not account for, and top of the list was ‘the person who has the illness’ (Engel, 1977, p. 131). Here the point is, at least, that biomedicine can theorize diseased or otherwise dysfunctional organs or systems, but has nothing to say, over and above that, about the person who has the illness. Equally, it can be added, cognitive psychological models of specific systems such as memory and attention, need a wider, person-level framework to theorize how lowered function affects the person, for example, or typically, by compromising agency.

To sum up, the cognitive revolution in psychology endorsed the relevance of mind to science by constructing causal explanatory models of behavior in terms of mental (or cognitive-affective) states. Within that overall framework, diverse psychology specialty areas focused on personal processes – beliefs, about the world and their own agency, personal goals, emotions, and behavior – in interaction with biological and social processes. These developments in psychology have wide implications and they surface again when considering biopsychosocial models, such as of impacts of social disadvantages on health, and of pain and service use, considered in Part 2, and models of clinical care, in Part 3.

The second aspect of post-dualism models mentioned above is that psychological processing is regarded as a function of, or implemented by, brain processing, hence merging psychology with neuroscience. Cognitive (or cognitive-affective) neuroscience (as the merger can be called) has developed alongside cognitive psychology (Albright, Kandel, & Posner, 2000).

The new post-dualist constructs of mind and body, further, accommodate crosstalk between neuroscience/psychology and biomedicine, in both directions. This is evident in the new fields of psychoneuroendocrinology (Fink, Pfaff, & Levine, 2012) and psychoneuroimmunology (Moraes, Miranda, Loures, Mainieri, & Mármora, 2018), as well as in specific models such as of chronic stress and pain to be considered later. These interdisciplinary research programs, involving neuroscience, psychology, and biomedicine, were inconceivable in mind–body dualism. They are examples of the rationale for expanding the BMM to the BPSM, in effect contributing content to the concept of ‘biopsychology’ or ‘psychological medicine’ within the BPSM.

A relatively new class of theories known as ‘embodied mind’, ‘embodied cognition’, or ‘4E cognition’, explicitly overturns dualism and are, therefore, potentially relevant to a revitalized BPSM. They are less familiar than theories discussed above, however, and for reasons of space I do not consider them here – for details of the theories and controversies, see e.g. Newen, Gallagher, and De Bruin (2018), Carney (2020), and for current applications in clinical psychology and psychiatry (e.g. Allen & Friston, 2018; Gjelsvik, Lovric, & Williams, 2018).

### Social determinants of health

Social factors can be accommodated within the conceptual framework of the new biopsychology because the social sciences have always employed comparable concepts, such as organization, rules and regulations, control (power), communication, and production and distribution of resources (e.g. Lasswell, 1936). In this sense, it is psychology and biology that made the theory changes critical to the BPSM, thereby becoming more aligned with concepts familiar in the social sciences.

The concept of socioeconomic status is closely connected to an individual's or group's access to resources, and the immediate relevance to health is that resources include what promotes good health (Bickel, Moody, Quisenberry, Ramey, & Sheffer, 2014; McGowan & Shahab, 2019). Over the past few decades, a substantial range of epidemiological studies have established that there are social determinants of health, that is, a positive correlation between higher social status and better health, the so-called social gradient in health, which underpins health inequalities (Marmot, 2006). This applies to both physical health and mental health (Bell & Marmot, 2022).

The resources that we need for biological health are well-known. Consistent with Schrödinger's insight into what life is, they include conditions of biological energy production. What we need for good psychological health is less well understood, probably because mind/body dualism never provided a useful definition of psychological life. In the new conceptualization of psychological life reviewed in the previous section, its conditions may be framed in terms of having sufficient agency (or autonomy). The extent of agency, like access to the conditions of physical well-being, depends on socioeconomic status. As Michael Marmot puts it: ‘The lower individuals are in the social hierarchy, the less likely it is that their fundamental human needs for autonomy and to be integrated into society will be met’ (Marmot, 2006, p. 1304). Autonomy is facilitated by social integration especially into dominant power structures, and conversely is downgraded by exclusion, by denial of voice, civil rights and protections, and other means of oppression. These issues have been explored mainly in and across feminist (Biana, 2020), postcolonial (Fanon, 1968), critical race theory (Delgado & Stefancic, 2001), and increasingly in Lesbian, Gay, Bisexual, Trans, and Queer (LGBTQ) literatures (Lee & Brotman, 2013). Possible pathways linking social factors to health outcomes will be reviewed below as examples of models of biopsychosocial causal mechanisms.

## BPSM research paradigms

### Investigating psychological and social impacts on health

Research designs relevant to the BPSM are those that examine the effects of psychological and social, as well as biological factors, on health outcomes (e.g. Lacombe, Armstrong, Wright, & Foster, 2019). Immediate findings are typically of correlations or associations, and control conditions of varying levels of stringency increase confidence in inference to causation. Large-scale group studies are necessary to identify small effects, and the methodology relies on statistical analytic methods such as regression that can estimate the effects of one or more variables on a health condition-dependent variable, estimating independent effects, and interactions moderating the effects of one independent variable by another. Multivariable regression models are applicable within the BMM, including biological variables only, but the expanded BPSM framework also accommodates inclusion of psychological and social variables, estimating their independent, additive, and interaction effects (e.g. Guloksuz et al., 2019).

BPSM compatible research studies were barely available when Engel proposed the new model in 1977. In fact, they began to appear at around the same time. The first clinical trials of psychological therapies appeared in the 1970s, heralding what has become a very large-scale research program of developing and evaluating psychological interventions for a wide range of health conditions and their complications. The early finding that cognitive therapy for depression was effective, and moreover, more effective than an antidepressant medication (Rush, Beck, Kovacs, & Hollon, 1977), reinforced the signal that the BMM was not enough, at least not for modeling and treating depression. At the same time, there was another major anomaly for the BMM, the emerging findings of social epidemiology, noted in the previous section, that social status affects a wide range of physical health and mental health outcomes, in the Whitehall Studies by Michael Marmot and colleagues (Marmot, Rose, Shipley, & Hamilton, 1978; Marmot et al., 1991).

The findings that are anomalous for the BMM but consistent with the BPSM are empirical data, related to specific influences on specific conditions at specific stages. There is nothing *a priori* in the empirical data. It is possible that a specific health condition at a particular stage may turn out to be primarily caused by only one kind of factor – biological, psychological, or social – and of course biomedical models of infectious diseases have had stunning successes in exactly this way. Such findings may be called a scientific-explanatory reduction to biological processes. This type of ‘reduction’ is different from theory-reduction of, for example, biology to physics and chemistry. Both types of ‘reduction’ are relevant to the relation between the BMM and the BPSM and both are in play in Engel's 1977 paper. The BMM would predict scientific-explanatory reduction to primary biological causes only across the whole of health, like the biomedical models of infectious diseases (or of effects of lesions or of genes of major effect). But this is an empirical, not a theoretical matter, and the emerging picture across health is that, especially for the noncommunicable diseases, and including all or practically all mental health conditions, the etiological picture is of at most a ‘patchy’ reductionism, and is more typically diverse, with multiple causal factors (Kendler, 2005, 2012).

Notwithstanding evidence of influence of psychological and social factors on health and disease, there remains a tendency, possibly attributable to long-standing reductionist assumptions in the science, to roll everything up into the biological. There are several examples of this option in the theoretical psychiatry literature. Samuel Guze's highly influential paper over 30 years ago, ‘Biological psychiatry – is there any other kind?’ (Guze, 1989) is an example, as indicated by the rhetorical nature of the title question. More recently, Peter White and colleagues proposed that mental disorders are brain disorders, without for a moment being unaware of the research showing the influence of psychosocial factors in the onset and course of many psychiatric conditions (White, Rickards, & Zeman, 2012). Likewise National Institute of Mental Health (NIMH's) Research Domains Criteria framework, which regards psychiatric conditions as disorders of brain circuitry (Insel et al., 2010).

To the extent that biology, neurology, and neuroscience are being broadly conceived to acknowledge the causal role psychosocial factors in some conditions, in etiology and course, and in prevention and intervention, these theoretical proposals are not reductionist and, albeit unhelpfully expressed, they are compatible with a broad biopsychosocial framework. However, proposals to roll the psychosocial up into the biological appear to be, in name, a kind of reductionism, so far by-passing the need to theorize the acknowledged causal role of psychosocial factors or biopsychosocial causal mechanisms.

### Theorized models of causal mechanisms

As well as findings of correlations (or associations) in well-controlled studies, the scientific picture benefits from also having a plausible theory that would explain apparent causal connections. Empiricism in science, relying on observation alone, controlled or otherwise, is well-known to be so far theory-free. In the present case of determining psychological and social causal connections, however, the problem has long been at exactly this point: the absence of a plausible theory of either psychological or social causation, still less theory as to how either could have material effects on biological processes, which were assumed determined by physics and chemistry alone. The assumed impossibility of psychological and social causation and the resulting downwards reductionist pressure inevitably encouraged skepticism toward any apparent empirical demonstration of the impossible. So while empirical evidence for psychosocial causes may accumulate, still, the theory problem is not yet solved and skepticism can persist.

Theory is necessary as well as data, of the sort outlined in the first part of the paper. In brief, psychological causation, implemented in brain processes, involves regulation of behavioral functioning toward attaining or maintaining some state. Social factors can causally interact with psychological processes, for example by regulating task demands and available resources. Psychological and social causal processes are both causal in the sense of regulatory, as is one kind of causation in biology, the other being energy transformations and exchanges covered by physicochemical laws. As to dysfunction, this has to involve disruption to regulation (however caused), because physicochemical laws cannot be disrupted. Models in which regulation/dysregulation are prominent are now to be found not only in biomedicine, but also in clinical psychology and psychiatry (Kendler & Woodward, 2021; Liu, Chua, Chong, Subramaniam, & Mahendran, 2020). Two well-known illustrations of theorized biopsychosocial causal mechanisms are given below.

The first example is a set of models of chronic stress, applied to epidemiological findings on the social determinants of health, aiming to explain pathways between unfavorable social status and unfavorable health outcomes. The key point for the present context is that the hypothesized causal mechanisms are biopsychosocial, indeed they have to be because the pathways implicated by the epidemiological research run across the three domains. Common hypothesized causal mechanisms and pathways include the following: low levels of social resources (e.g. working poverty and other forms of social exclusion) lead to chronic lack of control over salient outcomes, leading to chronic psychological stress, raising risk of anxiety and depression, while the chronic physiological arousal associated with chronic psychological stress raises the risk of dysregulation and damage across multiple biological systems and hence poor health outcomes. There is not space here nor is it the intention to review the large literature on chronic stress models (O'Connor, Thayer, & Vedhara, 2021; Roberts & Karatsoreos, 2021), but simply to give an illustration of a substantial research program on biopsychosocial causal mechanisms.

The second example of a new causal explanatory biopsychosocial theory with wide application comprises new models of pain perception, implicating neurobiological–psychological processing as well as peripheral physiological or structural damage. The new models implicate the person's negative appraisals of the meaning of pain and expected adverse effects on their lives and task demands, and associated Central Nervous System (CNS) pain-processing mechanisms (Garland, 2012; Ong, Stohler, & Herr, 2019).

This new understanding of pain perception is directly relevant to conditions dominated by pain, but there is a broader point that is relevant to the health sector as a whole, specifically to drivers of service use. The complex of pain, distress about pain, with associated impairment of functioning, is close to ‘feeling unwell’ and is a main driver of referral and service use. It is increasingly recognized that significant proportions of patients with such presentations turn out to have medically unexplained symptoms. ‘Medically unexplained’ here actually means *biomedically* unexplained, and biomedically orientated clinics typically have no biopsychosocial management protocols in place; consequently needs are not met, and the patient journey can be potentially long and costly; for example, in general practice and medical clinics (Jadhakhan, Lindner, Blakemore, & Guthrie, 2019), cardiology (Lenderink & Balkestein, 2019), neurology (Carson et al., 2003), and surgery for some pain presentations (Louw, Diener, Fernández-de-Las-Peñas, & Puentedura, 2017).

### Neuroscience and genetics are biopsychosocial

This overview of BPSM research paradigms with examples of major research programs has to briefly mention that the two life sciences that have accelerated the most in recent decades – genetics and neuroscience – are suited to a biopsychosocial theoretical framework. Indeed, it's more than that; they have been instrumental in making the new BPSM compatible core theory reviewed in Part 1.

It was advanced in genetics that introduced into biology theoretical ideas of a new kind of science involving coding, information-transfer, error, regulation and control, additional to energy-transfer and -exchanges covered by physical–chemical laws (equations). Further, theories of genetics have always been thoroughly interactional across domains, in evolutionary theory, and recently in the new field of epigenetics, including in psychiatry (Campanile, Fanelli, Fabbri, Serretti, & Mendlewicz, 2022; Cecil, 2020).

The same theory-shift that transformed biology also transformed neuroscience and cognitive psychology, enabling a coherent biopsychology. As to the domain of social interactions, there is no shortage of research programs on its major importance to our biopsychology in phylogenesis (Barrett, Henzi, & Barton, 2022) and ontogenesis (Blakemore, 2008).

## Clinical implications

### Clinical trials and guidelines

The clearest clinical implications of the BPSM, in contrast as always with the narrower BMM, is accommodation of psychological and social factors as well as biological factors relevant to clinical management and treatment. The importance of this broader scope has been substantially supported in the clinical trials literature, appearing mainly after Engel wrote his 1977 main paper. Large-scale clinical therapeutics research programs in the decades since have studied and shown the effectiveness of some psychological therapies for a large range of health conditions (Barkham & Lambert, 2021), of combination therapy, medication plus psychotherapy, for some conditions such as depression (Breedvelt et al., 2021), and of social treatments such as social support (Brown et al., 2020; Wang, Mann, Lloyd-Evans, Ma, & Johnson, 2018). The details of exactly what helps what are always specific to details of treatment, condition(s) and stage, but the overall picture that has emerged from the clinical trials literature is consistent with the broad biopsychosocial framework, in the precise sense that any narrower framework – envisaging treatments that are biological only, psychological only, or social only – omits some effective treatments for some health conditions at particular stages.

Now that there is a substantial clinical trials literature, summarized and adapted in clinical guidelines, it is of major importance in clinical decision making. And the broad message, as above, is that the broad biopsychosocial framework is required to accommodate it.

Like the BMM, the BPSM covers not only causes of onset (etiology) and treatments, but also post-onset maintaining causes that adversely affect prognosis. The models of chronic stress and of pain outlined above are examples of maintaining mechanisms across the biopsychosocial domains. Social determinants of health associated with chronic stress, for example, are typically on-going, affecting not only illness onset but also prognosis, for example, by adversely affecting access to treatment (Schneider, Roots, & Rathmann, 2021).

### Theorizing personal, interpersonal, and institutional factors in clinical care

A lot more is going on in clinical care than decisions as to what treatments to recommend, including personal, interpersonal, and institutional processes. Specific issues include the role of the person as patient – in determining what is wrong, whether anything is wrong, in collaborating on a treatment plan – the imperative of ‘compassionate’ care (Hodges, Paech, & Bennett, 2020), and institutional/professional factors supporting or jeopardizing good clinical care (Mannion et al., 2019). Engel says a lot of interesting things about all these things in his 1997 paper and others around that time (Engel, 1980, 1982), and they can be considered as part of what is covered by the BPSM.

While persons, interpersonal relations, and institutions can be accommodated within the BPSM, the contrast with the BMM is, as always, clear. These matters are simply *out of the scope* of the narrower BMM: they are not in its ontology, and therefore, it has no idea of them at all, still less their causes and effects. Therefore, adherence to the BMM (or to any model or line of thought, by whatever name, which regards biological processes alone as being causally relevant to health and disease) will have to construe these other matters in another way, broadly not as science, but as ‘art’. There is of course a grand tradition of this approach in medical theorizing – see, for example, Nassir Ghaemi (Ghaemi, 2010) – and Engel argued against it, favoring rather a broadly scientific-investigatory approach to understanding and improving, for example, receptive and expressive clinical communication skills, and institutional supports of clinical professional care (Bolton, 2020; Engel, 1978, 1980).

The revitalized, cross-disciplinary BPSM proposed here can be used to theorize personal and institutional factors relevant to clinical care and highlight their role as critical and not merely discretionary considerations. For example, the fundamental importance of personal agency in psychology is consistent with the central role of the person as patient, and the fundamental importance of socio-political factors in regulating recognition and access to resources can help theorize and highlight interpersonal, institutional, and wider political processes that affect clinical care.

The key added value of the BPSM, in contrast with BMM, is that it accommodates personal, interpersonal, and institutional factors in clinical care within the causal systems affecting health and disease. That said, it should be noted that these issues have been most theorized by other models of care, for example patient or person-centered models (Epstein & Street, 2011; Nolte, 2017), and the more recent Recovery Model (Hare-Duke, Ng, & Slade, 2022), and in reports and studies on healthcare institutional failure (Reader & Gillespie, 2013). There is the further important point that the increasing voice of the person as patient has been substantially a consequence of activism and wider socio-political movements, not a matter of healthcare theory and research (Brown, 1981; Rashed, 2019).

## Conclusion

Engel's proposal in the late 1970s that a new model was needed to take account of not only biological factors affecting health and disease, but also psychological and social factors, was made at a time when the theoretical and empirical backing for it was not established but was rather in construction. The proposed new BPSM can be regarded as being, at the time in the late 1970s, a general empirical hypothesis that psychosocial as well as biological factors are implicated in the causes and cures of illness, and as such, it could have turned out false. As things have turned out, however, the model as a general empirical hypothesis has been confirmed. The determination of relevant evidence in the intervening decades has required the development of new research methodologies capable of determining multifactorial influences on onset, course, complications, and treatments. The overall picture of causes and cures that has emerged, comprising specifics on many particular health conditions and treatments, is broadly biopsychosocial rather than narrowly biological, which is why the terms ‘biopsychosocial’ or ‘biopsychosocial model’ have established extensive application in the clinical literature and in healthcare classrooms. Regarding theory and mechanisms, Engel recognized that reductionism of various sorts in the basic sciences of biology and psychology stood in the way of conceptualizing biopsychosocial causation, and that radical new nonreductive theories were required. As outlined in this paper, these radical changes required to theorize the BPSM were in fact already in their early stages by the late 1970s and are now standard science. Empirical findings, new research paradigms, and theories developed in the last few decades effectively update and revitalize the BPSM.
